# IGA Antibody Induced by Immunization With Pneumococcal Polysaccharides Is a Prognostic Tool in Common Variable Immune Deficiencies

**DOI:** 10.3389/fimmu.2020.01283

**Published:** 2020-06-24

**Authors:** Federica Pulvirenti, Cinzia Milito, Filomena Monica Cavaliere, Ivano Mezzaroma, Francesco Cinetto, Isabella Quinti

**Affiliations:** ^1^Department of Molecular Medicine, “Sapienza” University of Rome, Rome, Italy; ^2^Department of Translational and Precision, “Sapienza” University of Rome, Rome, Italy; ^3^Department of Medicine—DIMED, University of Padova, Padua, Italy; ^4^Internal Medicine I, Ca' Foncello Hospital, Treviso, Italy

**Keywords:** common variable immunodeficiency, *S. pneumoniae*, pneumococcal polysaccharides vaccine, respiratory infection, specific IgA antibodies

## Abstract

The evaluation of the response to vaccination in patients with inborn errors of immunity is a tool to evaluate T-dependent and T-independent antibody residual function of B lymphocytes and it is part of the diagnostic definition for Common Variable Immune Deficiencies. Currently used classifications for Common Variable Immune Deficiencies patients are based on the frequency of B cell subsets, and have been proven as a valid instrument for identification of patients at higher risk of infectious and non-infectious complications. This 6-years period observational study delineated the measurement of specific IgA antibodies induced by a 23-valent pneumococcal polysaccharides vaccine by a standardized ELISA for the quantification of IgA antibodies to all 23 pneumococcal serotypes as an additional prognostic marker in 74 CVID patients. The inability to mount an IgA-mediated response against the pneumococcal polysaccharide antigens or the inability to maintain the antibody response over time identified poor IgA CVID responders with severe immunological impairment, great risk of co-morbidities, and poor prognosis. The division of CVID patient into specific IgA-non responders and IgA-responders discriminated better than other CVID classifications for infectious risk, while it overlapped for non-infectious complications. Our study suggested to add the evaluation of the antibody response by the 23-valent IgA assay in the clinical monitoring of CVID patients.

## Introduction

Immunoglobulin serum levels and antigen-specific antibodies are included as diagnostic criteria in the assessment of patient with inborn defects of immunity (1). A consensus definition for the diagnosis of Common Variable Immune Deficiencies, the most common symptomatic primary antibody defects, included evaluation of T-dependent and T-independent antibody responses at the time of diagnosis (2). In clinical practice, specific IgG measured in pre- and post-vaccination samples produced in response to pneumococcal polysaccharides immunization is the most used test to evaluate a T-independent antibody response (3). Antibody responses mediated by isotypes other than IgG are also a proper assay at diagnosis and during the course of the diseases in primary and secondary antibody deficiency patients who are under IgG replacement therapy (4, 5). Our group contributed to validate the measurement of the IgA and IgM response to all 23 pneumococcal serotypes (PnPS) present in the polysaccharide vaccine (Pneumovax®) in healthy donors (6), in a wide cohort of patients with Common Variable Immune Deficiencies (CVID) (4), and in children with Transient Hypogammaglobulinemia of Infancy (7).

Commonly used classifications for CVID patients are based on the frequency of B cell subsets. These immune-phenotypic classifications, and in particular the studies done by EUROclass group (8) and by the Freiburg group (9), take into consideration the frequency of peripheral B cells, class-switched memory B cells, and of CD21low B cells. These classifications have been proven as a valid instrument for identification of CVID patients at higher risk of infectious and non-infectious complications.

We conducted an observational study over a 6 years period in a population of CVID patients immunized by Pneumovax® to define if IgA-mediated responses to pneumococcal polysaccharides could be an additional prognostic marker in CVID patients. The results allowed to identify CVID patients with a more severe immunological impairment, a greater risk of co-morbidity, and a worse prognosis. The IgA response discriminates better than other classifications for infectious risk, while it overlapped for non-infectious complications.

## Materials and Methods

### Study Design

This single-center study was carried out in the Referral Center for Adult Primary Immune Deficiencies of Rome, Italy. Seventy-four CVID patients (age 49.1 ± 14.7 years, 33 males and 41 females) and 20 healthy volunteers (HD) (age 37.4 ± 16.1 years, 13 males and 7 females) were enrolled. All patients included in this prospective observational study over a 6-years period were diagnosed as CVID following the ESID/PAGID criteria (10). In particular, patients >4 years of age, had at least one of the following: increased susceptibility to infection, autoimmune manifestations, granulomatous disease, unexplained polyclonal lymphoproliferation, and affected family member with antibody deficiency; AND marked decrease of IgG and marked decrease of IgA with or without low IgM levels; AND at least one of the following: poor antibody response to vaccines, low switched memory B cells; AND exclusion of secondary causes of hypogammaglobulinemia; AND no evidence of profound T-cell deficiency. All patients were on IgG intravenous or subcutaneous substitutive therapy with a cumulative monthly dosage of 400 grams/kg with intervals between administrations individualized in order to keep IgG trough levels (TL) >600 mg/dl. All patients and HD were immunized with a single dose of a 23 pneumococcal polysaccharides vaccine (Pneumovax®). For CVID patients, demographic characteristics and clinical data were abstracted from the medical record, including the occurrence of infections, immunoglobulin levels at the time of diagnosis, IgG TL, presence of bronchiectasis identified by computed tomography scan, autoimmunity including cytopenias, inflammatory bowel disease, seronegative arthritis, thyroiditis, alopecia, vitiligo, primary biliary cirrhosis, sicca syndrome, and enteropathy defined as persistent chronic diarrhea, weight loss, steatorrhea, and malabsorption. During the 6 years follow up, concomitant CVID-related conditions and outcome, number of upper and lower respiratory infections, IgG TL, B and T cell subsets were recorded. All patients enrolled provided their written informed consent. The Ethical Board of the “Sapienza,” University of Rome approved this study (RIF.CE: 5325).

### Immunization

Patients and HD were immunized with 1 dose of Pneumovax® (Merck and Co., Inc., West Point, PA, Lederle Pearl River, NY, USA) a 23-valent polysaccharide vaccine which contains the pneumococcal serotypes 1, 2, 3, 4, 5, 6B, 7F, 8, 9N, 9V, 10A, 11A, 12F, 14, 15B, 17F, 18C, 19F, 19A, 20, 22F, 23,F and 33F. Based on our previous study (11) on the antibody response kinetics, blood samples of CVID patients were collected, aliquoted and stored at −20°C at the day pre-immunization (pre), and at 4 weeks post-immunization (post 1). A third assessment was done at 36 ± 6 months post-immunization (post 2). ELISA test was used to quantify the serotype-specific anti-PnPS IgA responses.

### ELISA for the Quantization of Anti-PnPS IgA

ELISA for the quantification of IgA antibodies to the 23 pneumococcal polysaccharide serotypes was done by an ELISA test (IgA VaccZyme™ ELISA, The Binding Site Group Ltd. (TBS), Birmingham, UK), with pre-coated microtiter plates with all 23 pneumococcal polysaccharide serotypes present in the Pneumovax vaccine. Absorption of interfering anti-cell wall polysaccharide (CPS) antibodies was incorporated in this assay. In a previous paper (4) we have addressed the issue on correlation between the 23-valent ELISA and the specific pneumococcal serotypes. We have already validated the IgA-mediated in a previous the paper (11). The antibody titers were calculated as previously published (4, 11).

### Immunophenotype

Flow Cytometry Analysis of peripheral blood mononuclear cells was performed with the combinations of 4 fluorochrome-labeled monoclonal antibodies, all obtained from BD Biosciences. The B-cell populations were identified as classical naïve (CD19+CD27-CD21+CD38+), switched memory (CD19+CD27+CD21+IgM–), IgM memory (CD19+CD27+IgM+IgD+), transitional (CD19+IgM++CD38++), CD21low (CD19+CD21-/lowCD38– B cells. Dead cells were excluded from analysis by side/forward scatter gating. FACS analyses were performed on a FACSCalibur instrument (BD Biosciences) using the CellQuest (BD) and FlowJo (Tree Star) software as we previously described (6). On the basis of the immunophenotype, CVID patients were classified as group IA, IB and II according to the Freiburg classification. The Freiburg classification identifies type I patients with severely reduced class-switched memory B cells (<0.4% of lymphocytes) and type II patients with class-switched memory B cells >0.4% of lymphocytes. Type I patients are subdivided into a group with expansion of CD21low B cells (type IA, > 20% of CD19 B cells) and a group with <20% of CD21 low B cells (type IB) (9). EUROclass classification defines CVID patients with <1% B cells as group B- and patients with more than 1% B cells as group B+. Group B+ patients are further divided into smB+ and smB- according to the frequency of switched memory B cells (>2% or ≤ 2% of total B cells). Dead cells were excluded from analysis by side/forward scatter gating (8).

### Statistical Analysis

Patient demographics and clinical characteristics are summarized by frequencies and percentages and means and standard deviations *(SD)* where appropriate. Comparisons of continuous parameters between treatment groups were calculated with a *t*-test if normally distributed and with a Mann-Whitney *U*-test if not normally distributed; differences in frequencies between groups were calculated by using the χ2 exact test. Time to event was calculated by Kaplan-Meier curves and expressed elapsed time to baseline Kaplan-Meier curves for CVIDs were split into two groups: (1) IgA responders; (2) IgA non-responders. The statistical significance was set at the conventional level of *P* < 0.050. All statistical analyses were performed using the statistical package Stata 11 (Stata Corp., College Station, Tex) and GraphPad7 (GraphPad software, San Diego, California, www.graphpad.com).

## Results

### CVID Patients Had an Impaired IgA Response to Pneumococcal Polysaccharide Vaccine

**Figure 1:**
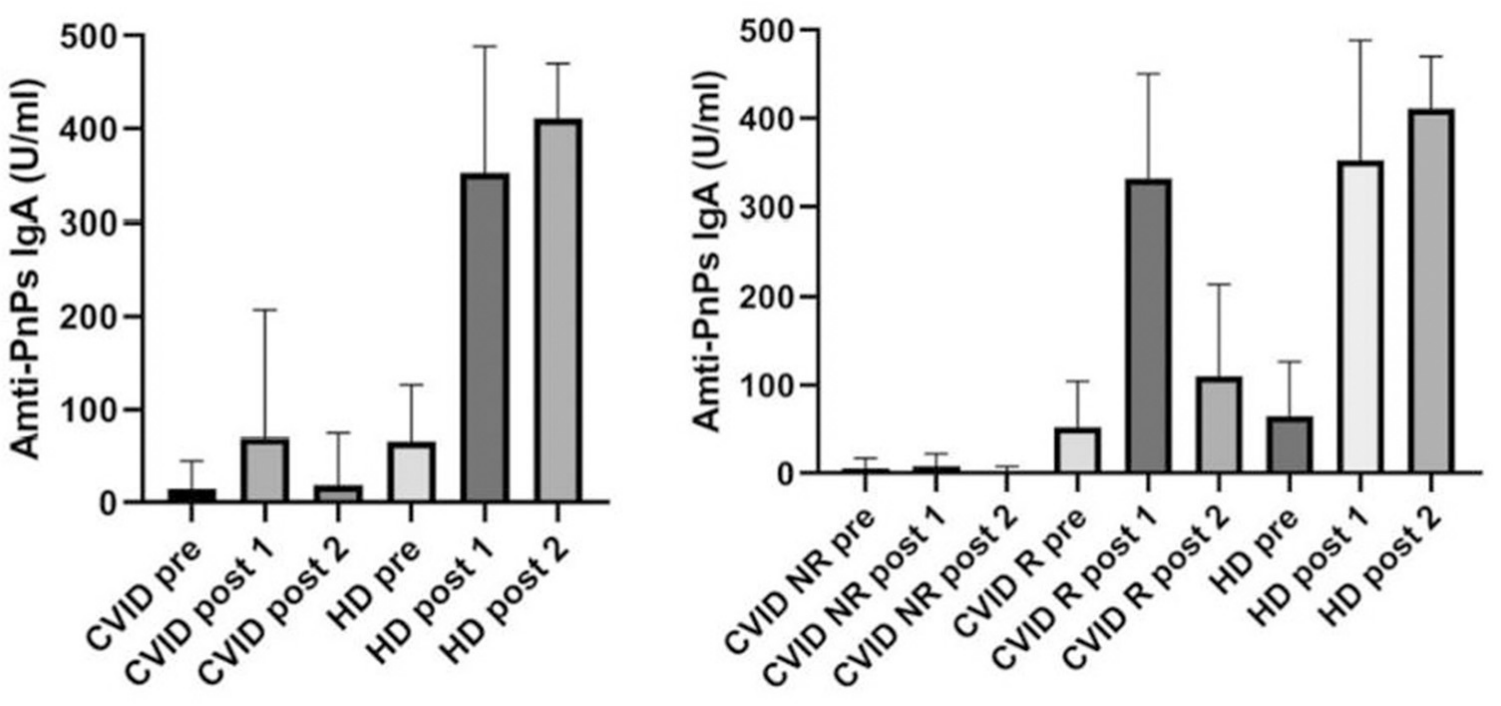
Specific PnPS IgA antibodies in healthy controls and CVID patients before vaccination (pre), 4 weeks after vaccination (post 1) and after 36 ± 6 months (post 2). CVID patients are classified as not responders (NR) and responders R according to IgA specific response.

Baseline characteristics of the 74 enrolled CVID patients are summarized in Table 1. We have already analyzed the IgA-mediated antibody response to the 23-valent polysaccharide vaccine (Pneumovax®) using a standardized ELISA 23 PnPS-IgA assay in healthy subjects (6). This standardized single-run procedure was based on a broad set of pneumococcus serotypes to measure the response to the 23 vaccine antigens present in the Pneumovax® vaccine. The kinetics of the IgA response to Pneumovax® showed a peak at 3–4 weeks after vaccination with an increase in PnPS-IgA antibody concentration of 10–12 times. The standardized ELISA 23 PnPS-IgA assay allowed to quantify the titer expressed as U/ml. The optimal cut off value for post-vaccination 23 PnPS-IgA antibody was determined at 80 U/ml (mean-−2SD). In this study, we evaluated specific IgA in HD and CVID patients before vaccination and 4 weeks later. Before vaccination, titers of anti PnPS IgA were 14.2 ± 30.7 U/ml, and 65.3 ± 61.2 U/ml in CVID patients and in HD, respectively. Four weeks post-immunization anti PnPS IgA titers were 69.2 ±138 U/ml, and 352.5 ± 136 U/ml in CVID patients and in HD, respectively. The cut off allowed to identify two groups of patients. Fourteen patients were IgA responders (IgA-R) and 60 IgA were non-responders (IgA-NR): IgA-R: 332 ± 118 U/ml and IgA-NR 6.4 ± 9.5 U/ml) (Figure 1). A second assessment was done 36 ± 6 months after the immunization in 63/74 patients (85%). All patients from the IgA-NR group were confirmed as being NR having no IgA anti PnPS IgA response (1.8 ± 5.7 U/ml). In the IgA-R group, nine patients were re-tested and five of them showed a long-lasting response (IgA-R: 201.8 ± 55.3 vs. HD: 280.3 ± 133.5 U/ml) (Figure 1) IgA R have a higher age than HD and CVID IgA NR. Higher response in older was nor related with previous exposure and to higher memory/recall response, since anti-pneumococcal polysaccharide responses decline with age (12).

**Table 1:** Characteristics of CVID patients at the enrollment.

	**All CVID** ***N*** **=** **74**		**IgA-NR** ***N*** **=** **60**		**IgA-R** ***N*** **=** **16**		**IgA-R vs. INR**
	**Mean**	***SD***	**Mean**	***SD***	**Mean**	***SD***	***P-value***
Age at the enrollment (years)	49.1	14.7	46.5	14.3	61.4	13.8	0.001
Age at diagnosis (years)	34.5	15.7	32.4	13.9	48.4	15.0	0.001
**IG level at diagnosis (mg/dL)**							
IGG	239.7	190.6	208.3	192.5	396.7	63.0	0.025
IGA	20.2	33.2	10.4	14.7	26.0	53.5	<0.05
IGM	22.2	25.3	17.3	18.2	34.8	39.7	<0.02
**IG at enrollment (mg/dL)**							
IGG	632.7	146.8	624.9	140.6	640.8	148.2	0.733
IGA	18.9	31.3	8.3	10.5	23.8	50.7	<0.05
IGM	20.5	23.3	14.1	14.6	35.2	37.1	<0.02

*All CVID patients are classified on the basis of anti PnPs IgA response as IgA non-responders (NR) and IgA responders (R)*.

### Frequencies of B and T Cells Subsets in IgA-R and in IgA-NR and Comparison With B-Cells Subset-Based CVID Classification

**Figure 2:**
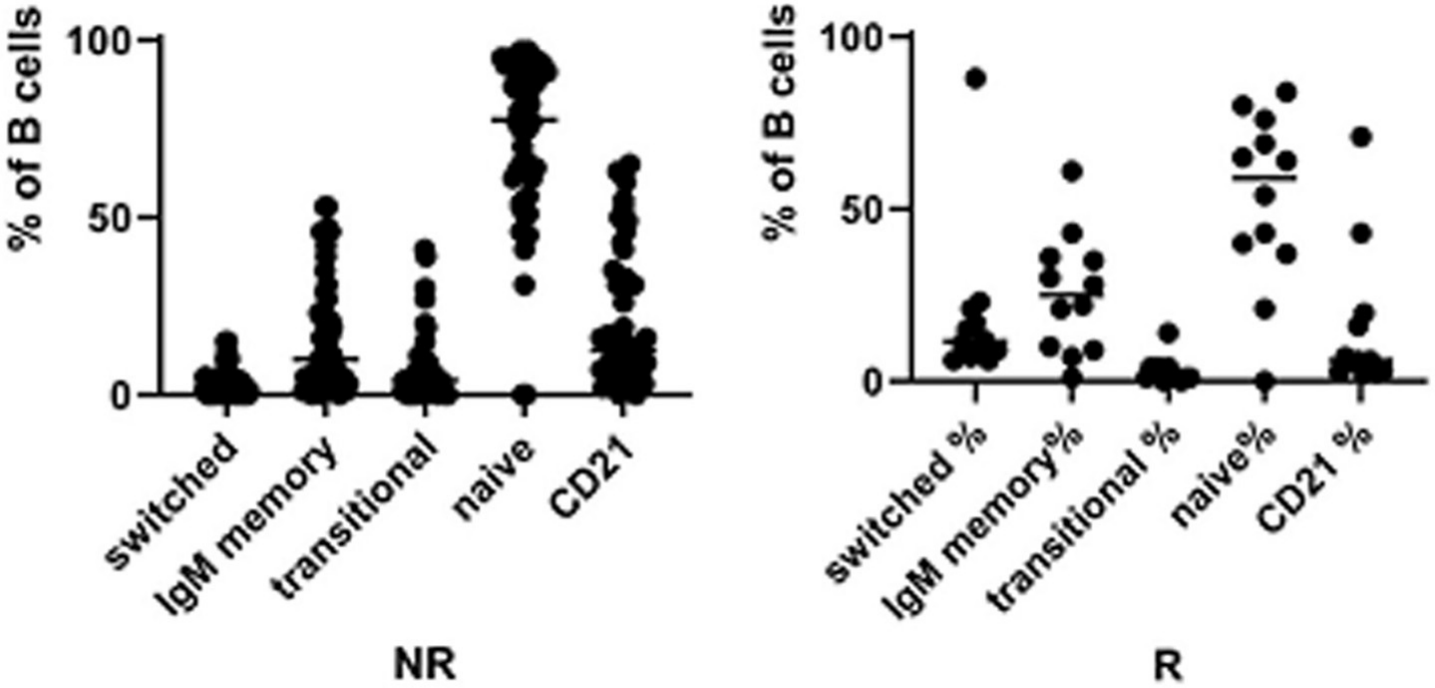
Frequencies of switched memory B cell, IgM memory B cells, Transitional B cells, naïve B cells and CD21low B cells in CVID IgA NR and CVID IgA R.

**Figure 3:**
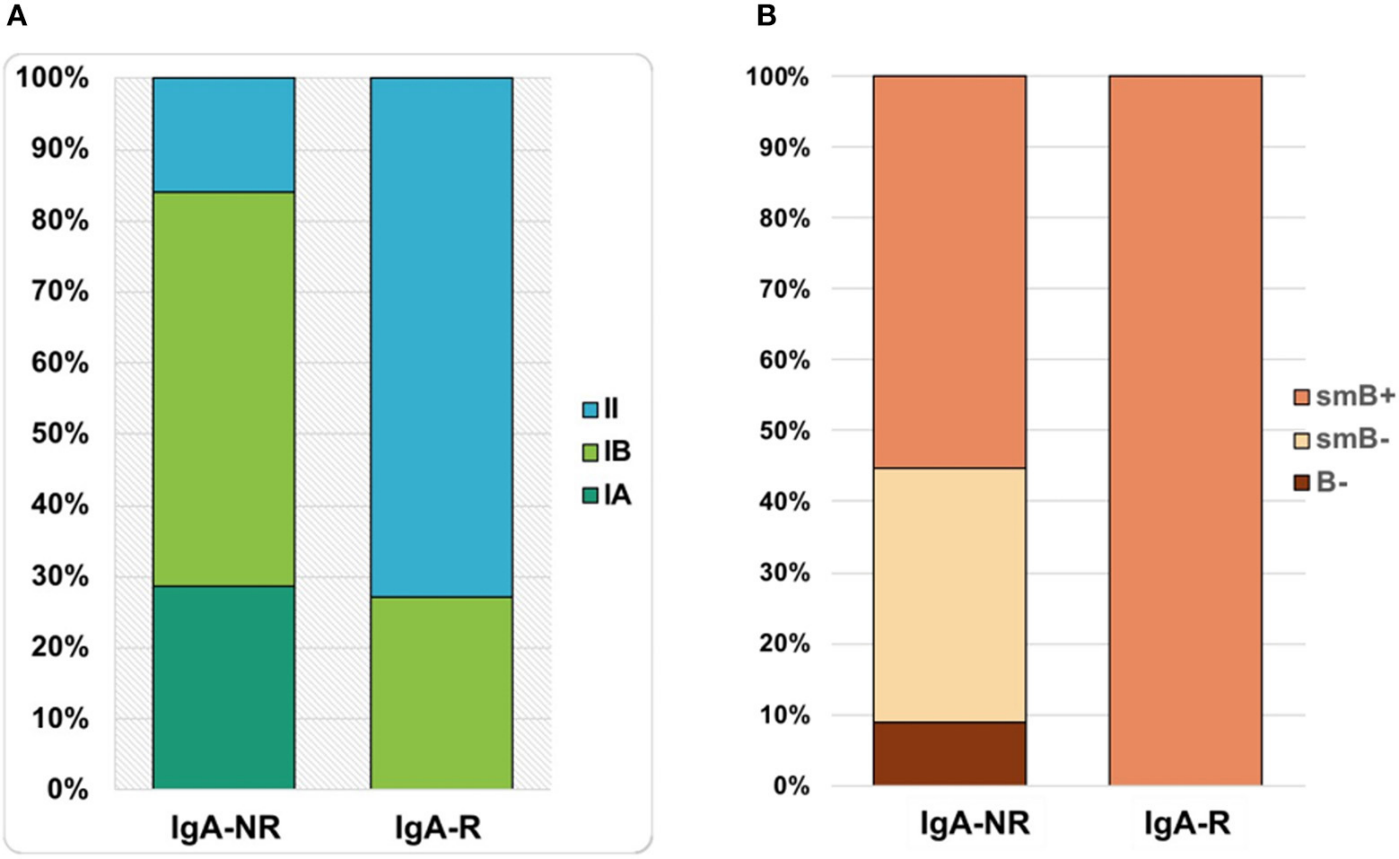
Percentages of patients classified as IgA-NR and IgA-R belonging to group IA, IB and II by FREIBURG classification **(A)** and to group smB+, smB-, and B- by EUROCLASS classification **(B)**.

The frequency of total B cells was similar within the groups. Patients from the IgA-NR group had an increased frequency of naïve B cells and a lower frequency of switched memory B cells compared to the IgA-R. The frequencies of marginal zone B cells, transitional B cells and CD21low B did not differ in the two groups (Figure 2). Frequencies and absolute count of B cells subsets and significance levels were summarized in Table 2. No difference was observed among T cell subsets frequencies in IgA-R and IgA-NR (Table 3). Based on B-cell subsets analysis, CVID patients were grouped following the Freiburg classification as group IA, IB and II. Interestingly, the IgA-NR phenotype included all the three Freiburg classes (IA: 28%, IB: 56%, II: 16%, whereas the IgA-R phenotype included only IB (27%) and II (73%) Freiburg classes. All IgA-R belong to the EUROclass smB+, while IgA-NR were B- (9%), smB- (36%), and smB+ (55%) (Figure 3).

**Table 2:** Frequencies and absolute count of B cells subsets and significance levels in patients grouped by post vaccination serum levels of Anti-PnPs IgA as IgA non-responders (NR) and IgA responders (R).

	**IgA-NR**		**IgA-R**		***P-value***
	**Mean**	***SD***	**Mean**	***SD***	
**B cells** **CD19+**					
% Lymphocytes	9.9	6.7	13.5	7.0	0.063
B cell/mm^3^	191.7	181.8	239.4	218.2	0.336
**Naive B cells** **CD27-IgM+IgD+**					
% B cells	73.4	22.9	57.5	20.2	0.010
B cell/mm^3^	140.7	145.2	123.4	92.6	0.489
**MZ B cells** **CD27+IgM+IgD+**					
% B cells	15.3	16.1	27.5	16.2	0.066
B cell/mm^3^	34.5	63.6	82.9	102.9	0.109
**Switched memory** **CD27+IgM-IgD-**					
% B cells	2.7	3.5	12.5	5.9	<0.0001
B cell/mm^3^	4.2	6.2	24.9	25.4	0.001
K/L ratio	1.5	0.6	1.3	0.1	0.051
**Transitional B** **CD38++IgM++**					
% B cells	7.2	10.4	3.2	3.9	0.165
B cell/mm^3^	12.0	25.5	5.1	3.8	0.386
**Plasmablasts** **CD38+++IgM±**					
% B cells	1.7	2.0	4.1	10.0	0.117
B cell/mm^3^	2.9	3.9	2.5	2.6	0.639
**CD21 low B cells** **CD21 low CD38 low**					
% B cells	21.6	19.0	10.3	12.3	0.368
B cell/mm^3^	37.5	50.4	50.5	108.5	0.057

**Table 3:** Frequencies and absolute count of T cells subsets and significance levels in patients grouped by post vaccination serum levels of Anti-PnPs IgA as IgA non-responders (NR) and IgA-responders (R).

	**IgA-NR**		**IgA-R**		***P-value***
	**Mean**	***SD***	**Mean**	***SD***	
Total T cells CD3+CD4-CD8-CD3+	76.2	9.5	73.2	7.5	0.286
% Lymphocytes cell/mm^3^	1352.8	679.5	1295.8	448.4	0.760
CD4 T cells CD3+CD4+	35.8	11.4	39.1	10.6	0.453
% Lymphocytes cell/mm^3^	598.8	358.8	630.8	241.8	0.880
CD8 T cells CD3+CD8+	37.6	12.0	32.4	10.1	0.207
% Lymphocytes cell/mm^3^	697.2	432.3	644.4	321.2	0.747
TCR alfa/beta CD3+TCRab+	86.5	9.8	87.7	9.3	0.702
% T cells cell/mm^3^	1168.4	625.9	1108.5	323.3	0.619
double negative T CD3+CD4-CD8-	1.8	1.2	1.6	0.8	0.431
% T cells ab cell/mm^3^	18.4	16.9	15.7	11.7	0.391
CD4 memory T CD4+CD45RO+	73.7	20.9	75.6	11.4	0.878
% CD4 cell/mm^3^	408.6	208.9	438.1	207.3	0.785
CD4 naive T CD4+CD45RA+	4.0	84.7	36.5	23.6	0.466
% CD4 cell/mm^3^	195.8	296.9	214.0	209.9	0.908
Late CD8 effector T CD8+CD27-CD28-	43.8	21.6	28.2	19.6	0.034
% CD8 cell/mm^3^	294.9	258.3	161.2	173.4	0.126
CD8 effector T CD8+CD27+CD28-	38.7	80.1	17.6	5.9	0.091
% CD8 cell/mm^3^	157.5	138.1	107.1	84.4	0.259
CD4Tregs CD4+CD25HCD127-	17.0	80.0	4.1	1.8	0.051
% CD4 cell/mm^3^	17.0	14.8	25.2	17.0	0.062
NK cells CD16+CD56+	8.3	6.6	8.0	6.2	0.701
% Lymphocytes cell/mm^3^	140.3	125.9	107.6	87.3	0.291

### Infectious, Non-infectious CVID-Complications, and Outcome

**Figure 4:**
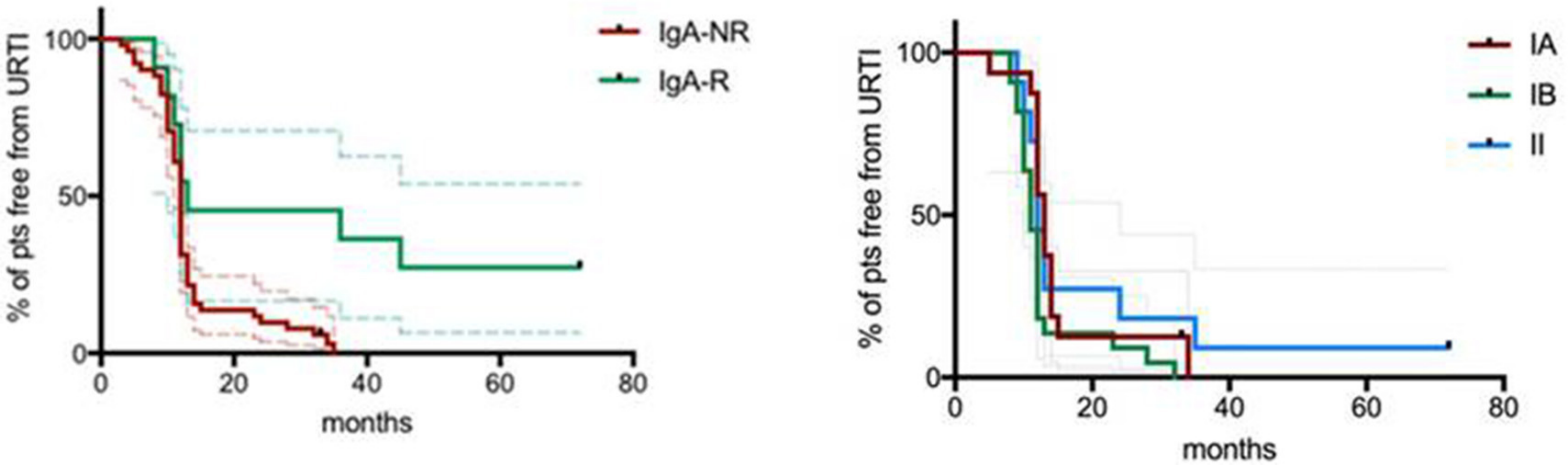
Percentage of CVID patients free from URTI grouped as IgA-NR and IgA-R (left panel) and percentage of CVID patients free from URTI grouped according to the Freiburg classification (right panel). URTI: upper respiratory tract infections. Dashed lines represent 5°th and 95°th interquartile.

**Figure 5:**
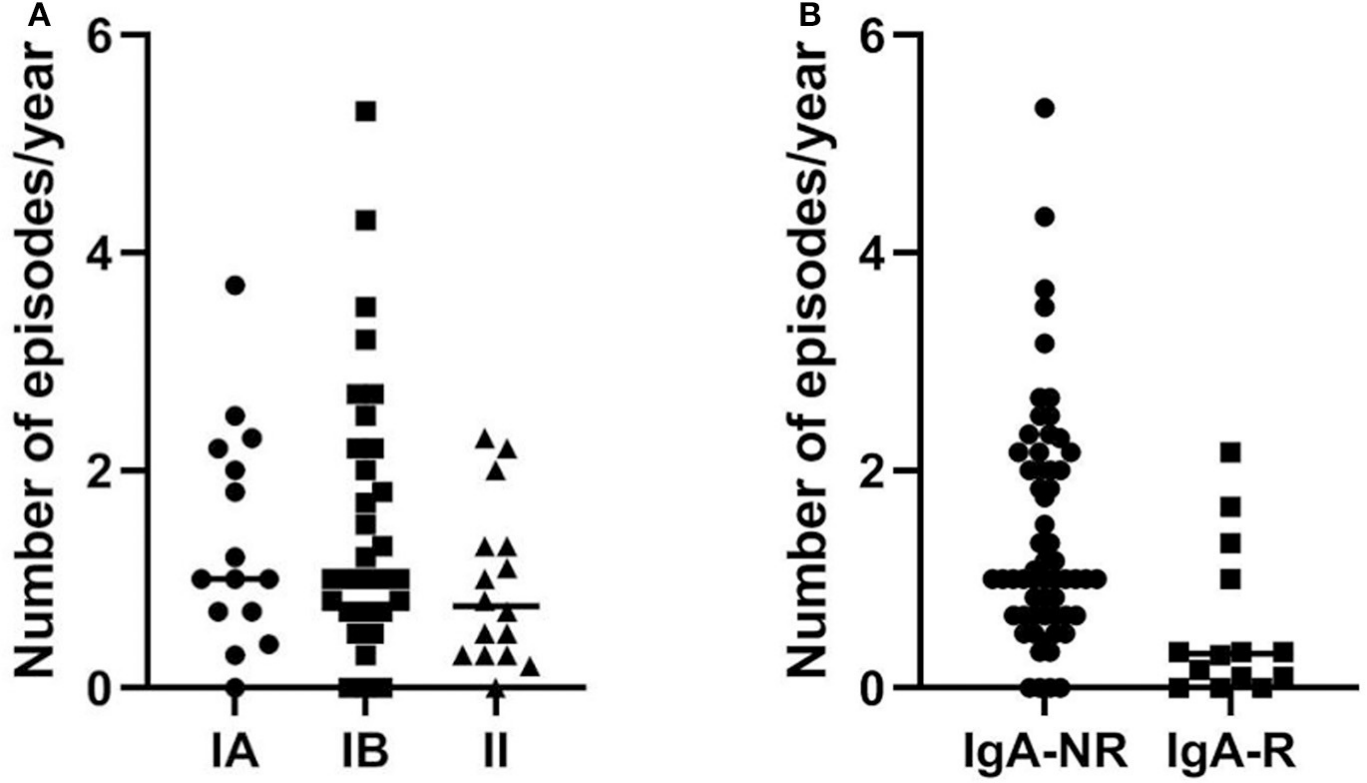
In **(A)** number of episodes/year of URTI in CVID patients grouped as group IA, IB e II according to Freiburg classification and in **(B)** number of episodes/year of URTI in CVID patients grouped as IgA non-responders (NR) and IgA responders (R) during the follow up. URTI, upper respiratory tract infections.

**Figure 6:**
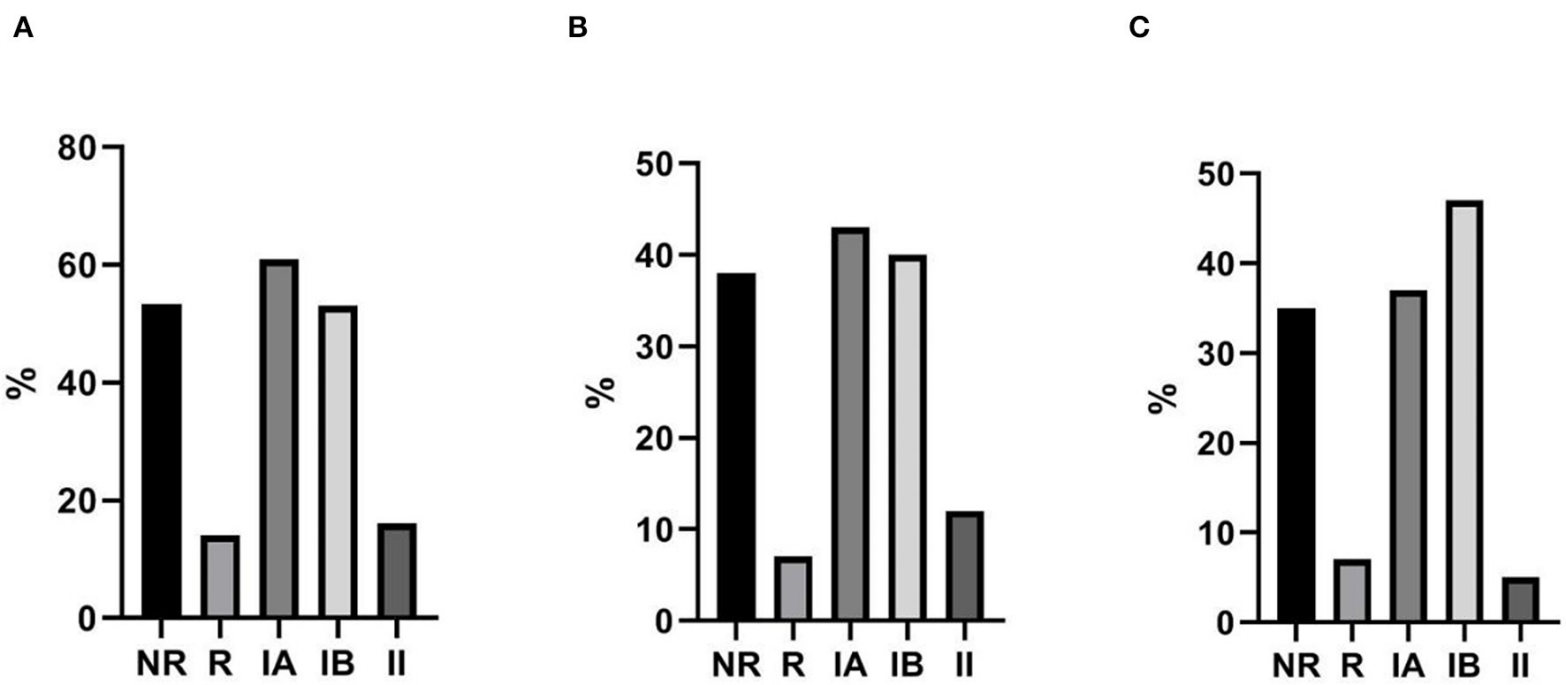
Non-infective CVID-related complications. Percentage of bronchiectasis **(A)**, autoimmunity **(B)** and enteropathy **(C)** in CVID patients grouped as IgA-NR, IgA-R, and IA, IB and II according to Freiburg classification.

The mean length of follow up (FU) for CVID participants was 64 ± 18.5 months. IgA-NR had a 2.8-fold higher risk to develop URTI in comparison to IgA-R (Log-rank *p* = 0.003; HR 2.85, 95% CI 1.4–5.7, Figure 4), with a higher rate of exacerbations (1,52 ± 1,28 vs. 0,92 ± 0,74 episodes per year, *p* = 0.013). We observed a similar number of episodes/year in IA group and in group IB, and a lower number of episodes in group II (IA 1.38 ± 1; IB 1.55 ± 1.27; II 0.93 ± 0.7, Figure 5). IgA-NR patients were also more prone to have LRTI (log-rank *p* = 0.009, HR 1.3, 95% CI 1.3–6.4, 0.5 ± 0.7 vs. 0.1 ± 0.3 episodes/year, *p* = 0.015). Likewise, a similar number of episodes/year were observed in IA group (0.7 ± 1) and IB group (0.5 ± 0.7) and a lower number in II group (0.2 ± 0.4). CVID patients are also prone to develop non-infectious complications. The prevalence of bronchiectasis was 53% in IgA-NR vs. 14% in IgA-R (*p* = 0.008, Figure 6A), and 61, 53, and 17% in group IA, IB and II, respectively. Moreover, the prevalence of autoimmunity was 38% in IgA-NR vs. 7% in IgA-R (*p* = 0.048, Figure 6B), and 43, 40, and 12.5% in group IA, IB, and II, respectively. The prevalence of enteropathy was 35% in IgA-NR vs. 7% in IgA-R (*p* = 0.049, Figure 3C), and 37.5, 47, and 5% in group IA, IB, and II (Figure 6C). Of the 74 subjects enrolled, 13 patients (15.5%) had died during the 6-years study time. Twelve out sixty patients (20%) of the IgA-NR group and 1/14 (7%) of the IgA-R group died. The mean age at death was 60.6 ± 15.6 years. Causes of death included lymphoid or other malignancies (five patients), infections (four patients), autoimmune cytopenia (one patient) and other not-CVID related conditions (three patients).

### Long-Lasting Response Patients

**Figure 7:**
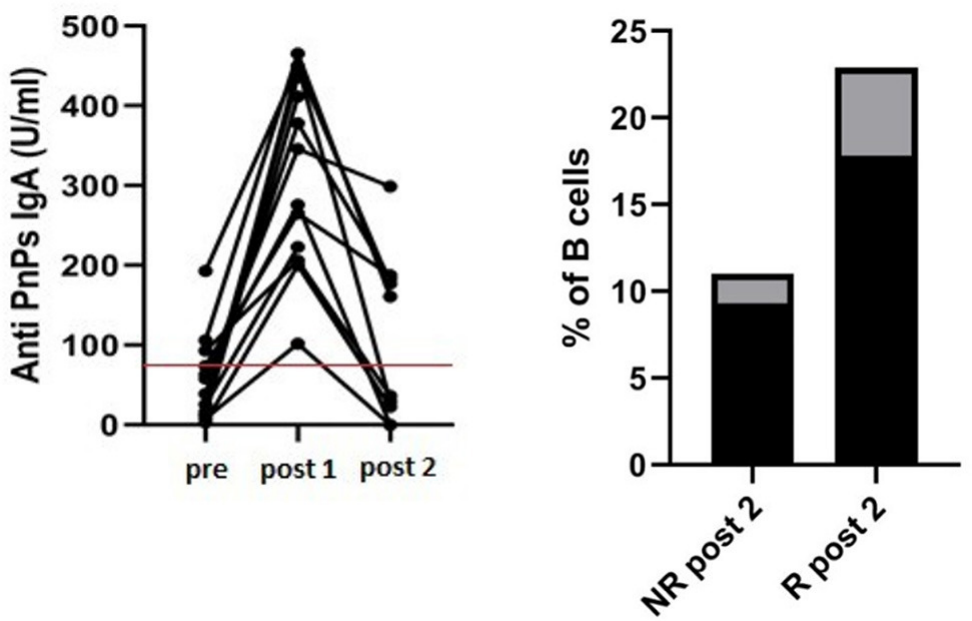
Long-term IgA antibody response in 16 CVID responders. Anti-PnPs IgA antibody in CVID before vaccination (pre), 4 weeks after vaccination (post 1) and after 36 ± 6 months (post 2) Red line indicates the cut off (80 U/ml) (left panel). Patients with a long-lasting IgA response at post 2 had a higher frequency of switched memory B cells in comparison to patients losing IgA response at post 1 (*p* < 0.02) (right panel). Black columns refer to mean value and gray columns refer to *SD* value.

At the second assessment (post 2), 36 ± 6 months after immunization, among IgA-R patients we identified a subgroup of five patients that showed a long lasting response with a level of specific anti PnPS IgA above the identified cut off (175 ± 13 U/ml) while 4 patients lost the specific IgA (19 ± 18 U/ml) (Figure 7). In the subgroup of long-lasting IgA-R we observed a higher frequency of switched memory B cells in comparison to IgA-R patients who lost IgA response after the first assessment (17.8 ± 5.1% of B cells vs. 9.3 ± 1.7% of B cells, *p* < 0.02).

## Discussion

In clinical practice, in the evaluation of the ability to respond to immunization induced by pneumococcal polysaccharide vaccine or polysaccharide-conjugated pneumococcal vaccine, the dosage of IgG specific anti-PnPS pre- and post-vaccination is usually used. The WHO has recommended considering an IgG cut-off ≥0.35 micrograms/ml of pneumococcal anti-serotype to each polysaccharide tested to evaluate the capacity of pneumococcal vaccines to give a post-vaccine protective response (13). The evaluation of the vaccination response in patients with inborn errors of immunity has a further informative role, being a tool to evaluate the residual function of B lymphocytes. However, studies to assess the B-cell function by their response to PnPS antigens may not be easily comparable if not the same set of pneumococcal antigen serotypes is used (5, 10). Moreover, anti-polysaccharide antibody responses are serotype-dependent, and fold increases are also dependent on the pre-vaccination titer in that high or very low titers might preclude the possibility to define the condition of responders or non-responders (14). In this study we selected the anti-pneumococcal polysaccharide response, since we have already shown the reliability and the reproducibility of this assay in patients with primary antibody deficiencies (4). It has also been demonstrated by our group that antibody responses mediated by non-IgG isotypes, and in particular the response mediated by IgA antibodies, can be considered a useful test to be performed at the time of diagnosis and during the course of the disease, particularly in patients with primary and secondary antibody defects undergoing substitutive therapy with IgG (5). We decided to analyses IgA antibody to polysaccharides since we have already previously demonstrated the associations of low serum total IgA levels with several CVID complications (15). Moreover, B lymphocytes can produce a large repertoire of IgA with T-independent mechanism useful in defense against pathogenic microorganisms and to reduce immune activation (16–18). In particular, IgA antibodies might control of invasive pneumococcal diseases (19).

In the immunocompetent subjects, for the measurement of the anti-PnPS IgA antibodies there are few data available in the literature concerning the reference values, and therefore the interpretation of the data on the efficacy of the antibody response can be difficult (20–22). It has been proposed to consider as a response an increase in pre-immunization titers of IgA PnPS antibodies specific for serotype of at least four times (23). Therefore, in our previous work, we analyzed the IgA-mediated antibody response to the 23-valent polysaccharide vaccine (Pneumovax®) using a standardized ELISA 23 PnPS-IgA assay in healthy subjects (4). This standardized single-run procedure measures the response to the 23 vaccine polysaccharide antigens present in the Pneumovax vaccine. The kinetics of the IgA response to Pneumovax® showed a peak at 3–4 weeks after vaccination with an increase in PnPS-IgA antibody concentration of 10–12 times. Later, we demonstrated that antibody responses mediated by non-IgG isotypes can be considered a useful test to be performed at the time of diagnosis and during the course of the disease in patients with primary antibody defects in a large cohort of patients with CVID undergoing substitutive therapy with IgG and in patients with Transitory Hypogammaglobulinemia of Infancy (5, 6).

In this study, we defined how the measurement of post-vaccine anti-PnPS IgA levels is a diagnostic parameter capable of classifying patients into groups with different risk of developing CVID-related complications over time. In particular, in the 6-years longitudinal study, we have evaluated the potential predictive value of the residual capacity to mount a specific IgA response. The inability to mount an IgA-mediated response against the pneumococcal polysaccharide antigens or the inability to maintain the antibody response over time was associated to a greater frequency of URTI and LRTI exacerbations due to a variety of pathogens (24, 25) during FU, to chronic lung damage and to a greater risk to develop non-infectious complications, autoimmunity and chronic diarrhea during the observation period. In contrast, CVID patients with IgA-mediated response had a reduced risk of clinical complications in the years following post-vaccination assessment. Fifteen per cent of enrolled patients died during the six-year study period and most of them belonged to the IgA-NR group.

In the classification of inborn errors of immunity, CVID is considered an heterogeneous clinical and immunological condition characterized by antibody defects (1). The wide range of clinical phenotypes in the CVID reflects the heterogeneity of immune defects associated with these diseases. Most of the classifications commonly used for patients with CVID are based on the determination of the frequency of B lymphocyte subpopulations. These immuno-phenotypic classifications consider the frequency of switched memory cells, and the number of peripheral B lymphocytes (EUROclass), and/or the frequency of CD21low B cells (Freiburg classification) to further stratify CVID patients with low number of switched memory cells. Both classifications have been demonstrated valid tools in the clinical setting. To validate our classification based on CVID IgA-R/IgA-NR we stratified our cohort according to the Freiburg and to the EUROclass classifications. Patients in IgA-R group showed higher frequency of switched memory and lower frequencies of CD21low B cells in comparison to the IgA-NR group. IgA-R belong mainly to the EUROclass smB+ group and to the Freiburg group II. However, our classification did not entirely overlap for the NR group. In fact, IgA-NR group patients belong to both the IA and IB classes, and 20% belong to class II (Freiburg), and to the smB– and smB+ classes and 10% to class B- (EUROclass). The correlation with clinical data between the classifications showed that our subdivision into IgA-R and IgA-NR could help better to discriminate on the rate of respiratory infections during the FU in comparison to the Freiburg or EUROclass ones. The three classifications (IgA-R/ NR, Freiburg class II and EUROclass smB +) showed a similar rate of other CVID-associated complications such as autoimmunity, bronchiectasis and chronic diarrhea.

In conclusion, the vaccine response to pneumococcal polysaccharide antigens by the standardized 23-valent IgA assay is easy to interpret. The evaluation of anti-PnPS23 IgA has been shown to be a prognostic marker, allowing to identify good and poor CVID responders. We suggest to add this test to identify patients with serious immunological impairment, a greater risk of co-morbidity and a worse prognosis who could benefit from closer clinical monitoring, and from additional preventive measures, including antibiotic prophylaxis (24). We suggest that antibody titers should be measured at ~4 weeks after immunization. If the response is adequate, titers should be measure again later on to check for the ability to maintain the antibody memory over time. A limitation of the study was not including children with PAD. However, IgA-specific pneumococcal polysaccharides in children with Transient Hypogammaglobulinemia of Infancy were analyzed in a previous paper (7) showing that the IgA response pre- and post-polysaccharide immunization was generally very low. Moreover, we suggested to validate the IgA anti-PnPS23 in other primary immune deficiency such as Selective Antibody Deficiency where the diagnosis is hindered by a lack of controlled clinical studies and the absence of a standardized definition of an insufficient pneumococcal polysaccharide antibody response (26).

## Data Availability Statement

The datasets generated for this study are available on request to the corresponding author.

## Ethics Statement

The studies involving human participants were reviewed and approved by Ethical Committee of Sapienza University. The patients/participants provided their written informed consent to participate in this study.

## Author Contributions

IQ, FP, and CM conceptualized the study. IQ, FP, CM, IM, and FC designed the protocol study. FMC performed all laboratory tests. FP, CM, IM, and FC recruited patients and collected data. FP and FC did the statistical analysis. IQ, FP, CM, and IM prepared the first draft of the manuscript. All authors reviewed the manuscript before publication.

## Conflict of Interest

The authors declare that the research was conducted in the absence of any commercial or financial relationships that could be construed as a potential conflict of interest.

## Abbreviations

CVID: Common Variable Immunodeficiency
PnPS: pneumococcal serotypes
HD: healthy volunteers
TL: trough levels
LRTI: lower tract respiratory infections
Sp: *S. pneumoniae*
URTI: upper respiratory tract infections.
